# Enrollment patterns among medicaid beneficiaries with sickle cell disease: Multistate findings from the sickle cell data collection program

**DOI:** 10.1371/journal.pone.0334883

**Published:** 2025-10-27

**Authors:** Ashima Singh, Mahua Dasgupta, Hannah K. Peng, Mei Zhou, Catie Clyde, Brandon K. Attell, Jhaqueline Valle, Sarah L. Reeves, Jeffrey Huebner, Angela B. Snyder

**Affiliations:** 1 Department of Pediatrics, Medical College of Wisconsin, Milwaukee, Wisconsin, United States of America; 2 Department of Pediatrics, Susan B Meister Child Health Evaluation and Research (CHEAR) Center, University of Michigan, Ann Arbor, Michigan, United States of America; 3 Georgia Health Policy Center, Andrew Young School of Policy Studies, Georgia State University, Atlanta, Georgia, United States of America; 4 Tracking California Program, Public Health Institute, Oakland, California, United States of America; 5 Department of Health Services, Wisconsin Medicaid Program, Madison, Wisconsin, United States of America; 6 Department of Family Medicine and Community Health, University of Wisconsin School of Medicine and Public Health, Madison, Wisconsin, United States of America; University of Illinois at Chicago, UNITED STATES OF AMERICA

## Abstract

**Background:**

Majority of individuals living with sickle cell disease (SCD) in the United States are enrolled in Medicaid. The objective of the study was to determine the patterns of Medicaid enrollment among individuals with SCD.

**Method:**

We determined the enrollment pattern among SCD Medicaid beneficiaries categorizing them in three groups: continuously enrolled, had exit and no return, had gaps in duration of enrollment during 2017–2019, leveraging the data from the Sickle Cell Data Collection Program in four states. We compared characteristics of individuals with gaps and those continuously enrolled using chi square tests.

**Results:**

Among 5883 children and 9260 adults, 70.5% and 61.8% respectively, were continuously enrolled. Gaps were observed in 12.5% of children and 12.9% of adults. A significantly smaller proportion of adults with gaps as compared to those who had continuous enrollment were disabled (CA:30.6% vs 65.3%; GA:23.7% vs 77.6%; MI:40.1% vs 69.5%; WI:39.8% vs 77.0%). Of all observed gaps, 60% were among adults. Enrollment patterns and gap duration varied by state.

**Conclusion:**

Approximately 12% of individuals with SCD have gaps in enrollment during our 3-year study period. Individuals with disabilities were more likely to have continuous enrollment. Future work is needed to determine reasons for observed gaps and their impact on SCD health outcomes.

## Introduction

Sickle cell disease (SCD) is the most commonly inherited blood disorder, affecting approximately 100,000 individuals in the United States, with the majority being African American or Black [1]. Data from the Sickle Cell Data Collection Program, a multi-state data collection effort, indicate that most individuals with SCD are enrolled in Medicaid [2]. Medicaid health insurance is essential in safeguarding the health of this vulnerable population in the United States. Medicaid is a joint federal and state program that helps cover medical costs for some people with limited income and resources. Each state in the United States of America runs its own program and thus eligibility criteria, programs, benefits and other policies can vary between states [3]. However, in general, most Medicaid beneficiaries, including those with SCD, are required to complete eligibility renewals, report changes in income and other circumstances, and otherwise respond to requests for eligibility-related information when the Medicaid agency identifies a need.

Individuals with SCD often have unpredictable painful crises, are at risk of chronic end organ damage and suffer from severe morbidity. In addition, they are likely to have several social economic stressors and live in areas with high social vulnerability [4]. Also, a large proportion of them are unemployed or inconsistently employed [5,6] which may limit their access to private insurance and at the same time impact their eligibility for Medicaid. The continuity of coverage and enrollment gaps for individuals with SCD enrolled in Medicaid is poorly understood.

Individuals may enroll, disenroll and then re-enroll in Medicaid insurance plans, which is often referred to as ‘churning’. Churning may occur for several reasons including short-term changes in income or circumstances that make them ineligible; both being barriers to maintaining continuous coverage [7]. Churning occurs in about 10% of Medicaid beneficiaries and less than half remain enrolled continuously for three years. Given that Medicaid eligibility is highly dependent on family income and disability status we expect more than 10% of Medicaid beneficiaries with SCD will have Medicaid enrollment gaps. Prior work shows that not all children enrolled in Medicaid have continuous coverage, even early in childhood, which may limit access to care [8]. However, the Medicaid enrollment patterns for individuals with SCD of all ages is unknown. We expect churning to be more frequent among adults, females, and non-disabled individuals as compared to children, males, and individuals with disabilities who have SCD. This study aims to determine the patterns of Medicaid enrollment, including continuity of coverage and duration of coverage gaps for individuals with SCD across four states.

## Methods

This is a retrospective cohort study leveraging data from the Sickle Cell Data Collection Program from four participating states. The Sickle Cell Data Collection Program, funded by the Centers for Disease Control and Prevention, collects health information to assess the long-term trends in diagnosis, treatment, and healthcare access for people with SCD in the United States health information about individuals with SCD living in the participating states [2]. While the specific data sources utilized in state-based programs differ by state, they commonly include data from newborn screening, administrative healthcare claims, and SCD specialty clinics operating within each state. The study population included individuals with SCD enrolled in Medicaid in CA, GA, MI, and WI for at least 1 month in 2017. An individual was determined to have SCD if they had a confirmatory newborn screening for SCD, reported with laboratory confirmation by a participating clinical site, or had three or more SCD-coded claims over a five-year period in any of the administrative data included in the respective states’ SCDC program [9,10]. Individuals in the study population were followed from the date of their first Medicaid enrollment in 2017 until the end of 2019 or the last available enrollment period. The characteristics of the identified cohort including their age, sex, race, ethnicity, region (metro vs non metro) were acquired from Medicaid. The metro/non-metro residence designation across all states was based on USDA 2013 Rural Urban Continuum Codes [11]. We also describe disability status and whether they were dual eligible or received partial coverage during the study period. The method to determine Medicaid disability status varied from state to state and is described in Table 1. For CA, aid codes were used to determine disability; GA and MI used flags for disability available within their data sources; WI applied a definition used by CMS in a prior analysis to determine disability [12]. Dual eligibility and partial coverage were determined based on indicators obtained from Medicaid data for all states.

**Table 1:** Disability definitions used by respective states in the study.

State	Method to determine the disability status	Values used
California	Aid codes	Aid Codes with “disability” as the eligibility category under which a person qualifies for Medi-Cal: 2H, 6H, 63, 64, 67, 6U, C3, C4, C7, C8, D4, D6, D7, 36, 6C, 6E, 6G, 6N, 6P, 6R, 6V, 6W, 6X, 6Y, 60, 66, 68, D5
Georgia	Aid category group	Blind and disabled
Michigan	Indicators in data	Either of the following criteria indicate a disability: CSHCS benefit plan or a program code for disability Or Age < 65 years and Medicare benefit plan or third-party liability
Wisconsin	Anyone > = 65 who is enrolled in a plan for only disabled people is considered as ‘Disabled’. If an individual is < 65 years of age AND is enrolled in a plan for only disabled individuals or that for disabled and ages 65 and older then we categorize them as ‘Disabled’	Plans considered to ascertain an individual as ‘Disabled’ If younger than 65 years: FC, MAP, MAPW, MCDW, QDWI, QMB, SLB, SLB + , SSIMA, TB, WCDC, IRIS, WCDH, WCDK, WWMA, MCD If 65 years or older: FC, MAP, MAPW, MCDW, QDWI, TB, WCDC, IRIS, WCDH, WCDK, WWMA

Enrollment patterns were described for the overall cohort and also by patient characteristics including sex, race, ethnicity, metro/non-metro residence, and their disability status.

## Analyses

Because of the differences in Medicaid eligibility policies across states and population groups, all analyses were stratified based on age groups. Federal laws require all states to cover children at least up to their 19^th^ birthday, assuming they meet income and other eligibility criteria [13]. Thus, in our study, beneficiaries age ≤ 19 years as of 12/31/2017 were considered pediatric and those age > 19 years as of 12/31/2017) were considered adults. The SCD beneficiaries were categorized into three groups based on their enrollment pattern:1) those who had no gaps/exits since first observed enrollment during the study period, 2) those who had one exit and no return, and 3) those with more than one exit and return in Medicaid. Those with one or more exits and returns were considered to have a ‘gap’ in enrollment. The duration of respective enrollment patterns is reported in months. We determined if there were differences in terms of age groups, sex and disability status between those with gaps and those continuously enrolled. The group with an exit and no return was excluded from these analyses, because we had no way to determine whether or not they had access to other forms of health insurance after exiting

We calculated the number of gaps per person and the duration of gaps for the SCD beneficiaries in each state. Further, we described the proportion of gaps <3 months (during which an individual may receive retroactive coverage). Only aggregate data were obtained and analyzed from each state. All groups were compared using a chi-square test. For all analyses, cell sizes less than 10 were suppressed and no statistical testing was done. If more than 20% of the population had missing information for a demographic characteristic, then only descriptive statistics were provided.

## Results

Our study included a total of 15,143 individuals with SCD who were enrolled in Medicaid for at least one month in 2017. Of these, 5883 (38.8%) were pediatric and 9260 (61.1%) were adult individuals; these percentages varied across states.

Pediatric: Of all the children included in the study (N = 5883), 49% were females, 86.9% were Black or African American, 95.0% were non-Hispanic/Latino individuals, and 92.1% lived in metro areas (Table 2). Among the pediatric beneficiaries with SCD, 45.9% were categorized as disabled in 2017 by the state-specific methodologies described above, only 7.2% had partial coverage plans and 1.3% were dually eligible for Medicare/Medicaid at some point during the study.

**Table 2:** Characteristics of children enrolled in Medicaid in 2017 across 4 states.

	Total (N = 5883)		CA (N = 1601)		GA (N = 2839)		MI (N = 1044)		WI (N = 399)	
	N	% (col)	N	% (col)	N	% (col)	N	% (col)	N	% (col)
**Gender**										
Female	2884	49.0	753	47.0	1412	49.7	524	50.19	195	48.87
Male	2999	51.0	848	53.0	1427	50.3	520	49.81	204	51.13
**Race** ^**a**^										
Black	3954	86.9	881	70.0	1762	93.3	956	93.8	355	0.93
Other	595	13.1	378	30.0	126	6.7	63	6.2	28	0.07
**Ethnicity** ^**b**^										
Hispanic	251	5.0	200	15.9	27	1.2	14	1.4	10	2.51
Not Hispanic/Latino	4749	95.0	1059	84.1	2277	98.8	1024	98.7	389	97.49
**Region** ^**c**^										
Metro	5421	92.1	1577	98.5	2436	85.8	1016	97.3	392	98.25
Non Metro/unknowns	462	7.9	24	0.25	403	14.2	28	2.7	7	1.75
**On plans for disabled individuals in 2017** ^**d**^										
Yes	2702	46.6	576	37.9	1139	40.1	810	77.6	177	44.36
No	3096	53.4	940	62.0	1700	59.9	234	22.4	222	55.64
**Dual eligible (study period: 2017–2019)** ^**e**^										
Not dual eligible at any point during the study period	5805	99.6	≥11	–	≥11	–	≥11	–	≥11	–
Dual eligible, yes at any point during the study period	21	0.003	<11	–	<11	–	<11	–	<11	–
**Benefit type (study period: 2017–2019)** ^**e**^										
Partial coverage, yes at any point during the study period	421	7.2	93	6.02	16	0.6	298	28.5	14	3.5
No partial coverage during the study period	5405	91.9	1451	94.0	2823	99.4	746	71.4	385	96.5

a: Race missing for 342 individuals from CA, 951 individuals from GA, 25 from MI, 16 from WI

b: Ethnicity missing for 342 individuals from CA, 535 individuals from GA, 66 from MI

c: Non metro counties are combined with unknown, out of state to mask all values <11

d: Disability determination unknown for 85 individuals from CA

e: Dual eligibility and Benefit type missing from 57 individuals

Adults: Of all adults included in the study (N = 9260), 63.6% were females, 89.6% were Black or African American, 97.3% were non-Hispanic/Latino, and 94% lived in metro areas (Table 3). Among adults with SCD, 58.9% were categorized as disabled in 2017, 33.2% were dually eligible for Medicare/Medicaid and 17.6% had partial coverage at some point during the study.

**Table 3:** Characteristics of adults enrolled in Medicaid in 2017 across 4 states.

	Total (N = 9260)		CA (N = 3574)		GA (N = 2609)		MI (N = 2416)		WI (N = 661)	
	N	% (col)	N	% (col)	N	% (col)	N	% (col)	N	% (col)
**Gender** ^**a**^										
Female	5887	63.6	2267	63.4	1713	65.7	1513	62.6	394	59.6
Male	3372	36.4	1307	36.6	896	34.3	902	37.3	267	40.4
**Race** ^**b**^	0									
Black	7321	89.6	2568	80.5	1978	97.2	2189	93.8	586	88.7
Other	853	10.4	621	19.5	58	2.8	145	6.2	29	4.4
**Ethnicity** ^**c**^										
Hispanic	233	2.7	188	5.9	9	0.4	21	0.9	15	2.3
Not Hispanic/Latino	8330	97.3	3001	94.1	2292	99.6	2391	99.1	646	97.7
**Region** ^**d**^										
Metro	8616	94.0	3492	99.4	2116	81.3	2366	99.0	642	97.1
Non Metro	550	6.0	20	0.6	488	18.7	24	1.0	18	2.7
**On plans for disabled** **individuals in 2017e**										
Yes	5455	61.1	1889	58.2	1629	62.4	1483	61.4	454	68.7
No	3477	38.9	1357	41.8	980	37.6	933	38.6	207	31.3
**Dual eligible** **(study period: 2017–2019)**^**f**^										
Dual eligible, yes at any point during the study period	3012	33.2	968	28.6	1004		783	32.4	257	38.9
Not dual eligible at any point during the study period	6059	66.8	2417	71.4	1605		1633	67.6	404	61.1
**Benefit type** **(study period: 2017–2019)**^**f**^										
Partial coverage, yes at any point during the study period	1599	17.6	565	16.7	523	20.0	464	19.2	47	7.1
No partial coverage during the study period	7490	82.4	2820	83.3	2086	80.0	1952	80.8	614	92.9

a: Gender information missing for 1 individual from MI

b: Race missing for 385 individuals from CA, 573 from GA, 82 from MI, 46 from WI

c: Ethnicity missing for 385 individuals from CA, 308 individuals from GA, 4 individuals from MI

d: Region missing for 62 individuals from CA, 5 from GA, 26 from MI, 1 from WI

e: Disability determination unknown for 328 individuals from CA

f: Dual eligibility and Benefit type missing from 189 individuals from CA

### Enrollment patterns

**Fig 1:**
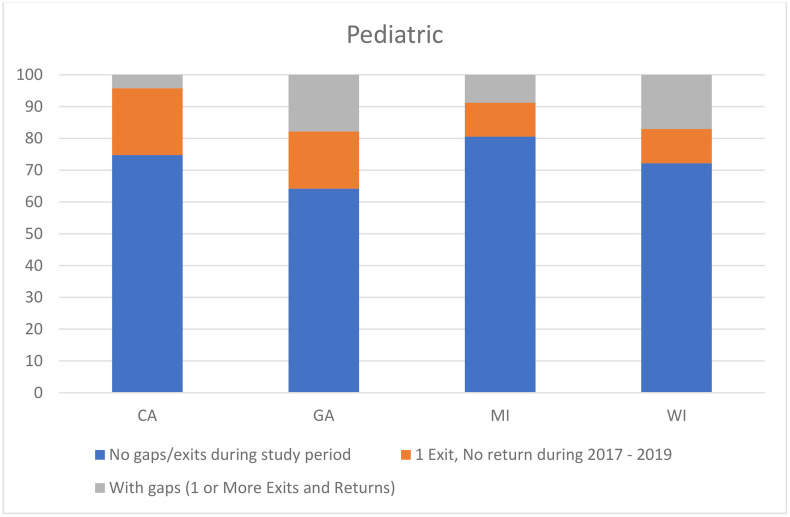
Proportion of pediatric SCD beneficiaries with continuous enrollment since first observed enrollment in 2017, 1 exit and no return, and with gaps within the states contributing data to this study.

Overall, a greater proportion of children as compared to adults were in the continuously enrolled group. However, the distribution of enrollment patterns across population groups varied by state, likely due to differences in eligibility policies. Among children, 70.5% were continuously enrolled from the first month in 2017 through the end of 2019, 17% had an exit and no return, and 12.5% had gaps during the study period. Fig 1 shows the enrollment patterns for the pediatric beneficiaries for each state. Of those who had an exit, an average of 43.6% of children reenrolled during the 3-year observation period. There were no significant differences in proportion of young children (0- less than 13 years) and teens (13–19 years) between the continuously enrolled group and those with gaps in any of the states. There were no significant differences in the gender distribution between those with continuous enrollment and those with gaps in CA, MI, WI (Supplement S1 Table). In GA, a significantly higher proportion of those with gaps were females as compared to those with no gaps (56.1% vs 48.6%, p = 0.003). A significantly lower proportion of children with gaps were disabled as compared to those with no gaps in GA (15.6% vs 50.4%, p < 0.0001) and MI (53.3% vs 81.2%, p < 0.0001). In CA, the same trend was observed, however, no p-values reached statistical significance. These results were masked for WI due to cell sizes <11.

**Fig 2:**
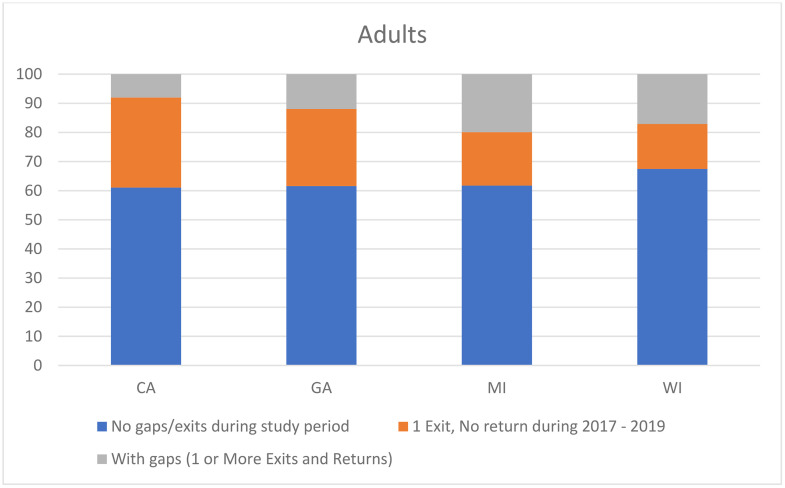
Proportion of adult SCD beneficiaries with continuous enrollment since first observed enrollment in 2017, 1 exit and no return, and with gaps within the states contributing data to this study.

Among adults, 61.8% were continuously enrolled, 25.3% had an exit and no return, and 12.9% had gaps during the study period. Fig 2 shows the enrollment patterns for the adult beneficiaries for each state. Of those who had an exit, an average of 39.1% of adults reenrolled during the 3-year observation period. There were no significant differences in the gender distribution between those with continuous enrollment and those with gaps in CA or MI (Supplement S2 Table). In GA, a significantly higher proportion of those with gaps were females (81.4% vs 63.0%, p < 0.0001) whereas in WI, a significantly lower proportion of those with gaps were females (51.3% vs 64.6%, p = 0.008) compared to those with no gaps. Across all four states, a significantly lower proportion of adults with gaps were disabled versus those with no gaps (CA: 30.6% vs 65.3%, p < 0.0001; GA: 23.7% vs 77.6%, p < 0.0001; MI: 40.1% vs 69.5%, p < 0.0001; WI: 39.8% vs 77.0%, p < 0.0001).

### Gap characteristics

Pediatric: There were a total of 851 coverage gaps experienced by 734 children across the 4 participating states during the study period. The mean (standard deviation) duration of these gaps was 6.6 (5.0) months in CA, 4.3 (4.7) months in GA,5.3 (5.7) months in MI and 3.4 (4.2) months in WI. Overall, 56.7% of the gaps were <3 months in duration. Supplement S3 Table includes the rate and proportion of gaps <3 months long among the pediatric SCD population in each state. The number of gaps per pediatric SCD beneficiary ranged from 1.10–1.29 months across the four states.

Adult: There were a total of 1489 coverage gaps experienced by 1190 adults across the 4 participating states during the study period. The mean (standard deviation) of gap duration among adults with SCD was 5.6 (5.3) months in CA, 5.5 (5.5) months in GA, 5.3 (5.2) months in MI, and 4.2 (4.7) months in WI. Of all gaps, 45.1% were <3 months long.

Supplement S4 Table shows the rate and proportion of gaps <3 months among the adult SCD population in each state. The number of gaps per adult SCD beneficiary ranged from 1.19–1.53 months across the four states.

## Discussion

Medicaid is the major provider of health insurance for individuals with SCD, and thus critical to ensuring continuous access to health care for this population. Our results show that across the states, ~ 12% of children and adults with SCD experienced churning, that is they had one or more exits and returns to Medicaid during the 3-year period of our study from 2017–2019. Overall, children were more likely to be continuously enrolled than adults.

There are only a limited number of studies looking at enrollment patterns beyond a 12-month period among the general Medicaid population. Our study reflects that there are 70% of children and 60% of adults who remain continually enrolled from 2017–2019. Some potential reasons for children enrolled in Medicaid to lose coverage include increase in household income that exceeds Medicaid’s eligibility threshold, failure to receive or properly complete and submit the insurance renewal paperwork on time, or if the parents or the state mistakenly drop coverage for the child when parents lose coverage. Adults with SCD may be unemployed, in and out of employment, and may not meet Social Security Administration or Medicaid agency requirements for disability [14] and hence loose coverage. Further, there are periods such as during pregnancy which may result in an individual being eligible for Medicaid only for a short duration. The loss of coverage for a chronic disease population with high rates of acute care utilization can impose substantial burden on the individuals and healthcare system. A previously published report shows that approximately 46% of the general public enrolled in Medicaid in 2005 continued to be enrolled through the end of 2007 [15]. The higher proportion of continuously enrolled people with SCD as compared to the general population may be due to a large proportion of individuals with SCD being eligible due to disability. Another report, more comparable to the time frame of our study, found that 8% of beneficiaries who disenrolled in 2018 re-enrolled within 12 months [16]. In our study, 14.6% of individuals were enrolled for less than 12 months which includes individuals with exits and no return or coverage gaps. This shows despite having a complex chronic condition, individuals with SCD still experience a substantial burden of gaps in their health coverage over time.

Every state operates its own Medicaid program within federal guidelines. The federal guidelines are broad and provide a large degree of flexibility in designing and administering the programs [17] which results in varied eligibility criteria and enrollment policies across states. This may be part of the reason for why we observe that a significantly higher proportion of females with gaps in GA. As a state that has neither expanded Medicaid (e.g., CA and MI) nor has a waiver program for childless adults (e.g., WI), in GA, most adult males qualify through disability, which tends to result in longer enrollment periods. Females, including older teenagers, may qualify through pregnancy or family Medicaid, which are more susceptible to churn due to income and condition-based eligibility. However, all states must provide Medicaid coverage for mandatory groups including low-income children and pregnant women, people with disabilities who receive Supplemental Security Income, and some low-income seniors. It is important to note, that our study was conducted after the Affordable Care Act was implemented, which had substantial impact on Medicaid plans and processes and expanded Medicaid eligibility to include adults without dependents. Those states who haven’t expanded Medicaid under ACA or a waiver have fewer eligibility pathways for adults without dependent children. Among the states contributing data to the study, two have adopted the Affordable Care Act’s (ACA) Medicaid expansion (CA, MI) and two have not (GA, WI), reflecting the state decision to adopt this expanded eligibility pathway for adults. WI uniquely covers adults without dependents up to 100% of the federal poverty level through a waiver [18]. Although, we did not aim to compare differences in coverage and enrollment pattern by state, the coverage differences may partly explain the variability in the characteristics of Medicaid beneficiaries with SCD across the states. Future work comparing enrollment patterns between states with expanded Medicaid coverage and those without expanded coverage may inform policies. However, irrespective of the underlying programmatic differences, our findings that individuals with continuous coverage were more likely to be determined disabled, highlights the interconnection between an individual’s health and functional status and their ability to maintain Medicaid coverage. However, the listing criteria to qualify for Social Security Disability for children and adults with SCD are stringent [14], and exclude many individuals with SCD who may have chronic debilitating complications. It is important to note, irrespective of disability status, all individuals with SCD require comprehensive and ongoing care to manage their condition and prevent complications.

Prior studies, although not specific to the SCD population, show that people who have gaps in their insurance coverage can have delays in preventive screening, higher rates of hospitalization, and high expenditures [19], whereas continuous eligibility in Medicaid is shown to be associated with better health outcomes [20,21]. There are no studies specific to SCD population that look at the impact of gaps in duration of coverage on health outcomes. Administrative claims data is limited to include healthcare service utilization during insurance coverage, however linking these data with statewide hospital association data can help compare acute care utilization rates between those with continuous coverage and those with gaps. Future work is needed to understand the impact of gaps or limited coverage duration on the health of individuals with SCD.

Apart from the impact of coverage gaps on health outcomes, managing the process of eligibility checks and re- enrollments can be an administrative burden to Medicaid enrollees. During the COVID-19 public health emergency as per the Families First Coronavirus Response Act (FFCRA) passed by Congress in 2020 [22], eligibility redeterminations were halted and continuous enrollment was granted to all enrollees. In 2023, the process of ‘unwinding’ this policy resumed across the nation. Although, the impact of the Act and its unwinding on access to healthcare for individuals with SCD is unknown, it is likely that the enrollment patterns and gaps in coverage will return to pre-COVID levels. This study helps establish the enrollment patterns among beneficiaries with SCD pre COVID-19, and these data will provide a baseline assessment to determine the impact of unwinding the FFCRA policy on individuals with SCD.

The complex scenario of high social vulnerability and unstable employment for those with a high-cost, chronic condition such as SCD warrants a disease-specific program with more flexibility to avail state benefits. One such example would be the WI chronic disease program for chronic renal disease, adult cystic fibrosis and hemophilia programs, that can cover individuals with the specific chronic conditions if not covered by Medicaid [23]. Stability in insurance coverage can reduce the public costs and total US healthcare expenditure, which is one of the highest in the world [24]. Continuous coverage would ensure that individuals with SCD receive the right care at the right time to keep them healthy. This is especially imperative in light of new expensive treatments such as gene therapy. As gene therapy becomes accessible to individuals with SCD, continuous coverage irrespective of additional eligibility criteria is needed to ensure that a potentially curative and life-transforming therapy is available to all who qualify based on their disease progression and does not lead to financial toxicity [25]. Further, continual coverage may provide a more comprehensive picture of what has been done historically to document their candidacy for gene therapy. Finally, our study informs the interpretation of findings from health services studies that are limited to people with continuous enrollment. These studies likely include a higher proportion of people who are disabled and are likely skewed towards those who are at risk of worse outcomes.

Our study has a few limitations. Our findings may not be generalizable to other states. Our analysis is restricted to Medicaid claims and enrollment data; therefore, we do not know if people transitioned to private insurance or were uninsured during their coverage gaps or after exiting Medicaid coverage. Aggregate data limits the ability to do individual level modeling.

## Conclusion

Despite having a chronic condition, 12% of individuals with SCD have gaps in Medicaid coverage during our 3-year study period. Across all states, individuals determined disabled were more likely to have continuous enrollment. Of all observed gaps, 40% were among children and 60% among adults. Future work is needed to determine the reasons for these gaps, and their impact on health outcomes. Also, future work is needed to determine the impact of FFCRA and the consequent unwinding on individuals with complex, chronic conditions like SCD.

## Supporting information

S1 Table Comparison of characteristics of pediatric SCD Medicaid beneficiaries continuously enrolled and those with gaps the years 2017–2019. (DOCX)

S2 Table Comparison of characteristics of adult SCD Medicaid beneficiaries continuously enrolled and those with gaps the years 2017–2019. (DOCX)

S3 Table Gaps in Medicaid enrollment among children with SCD. (DOCX)

S4 Table Gaps in Medicaid enrollment among adults with SCD. (DOCX)
