# Supplementary material for: Enrollment patterns among medicaid beneficiaries with sickle cell disease: Multistate findings from the sickle cell data collection program
Source: PLoS One. 2025 Oct 27;20(10):e0334883. doi: 10.1371/journal.pone.0334883 (PMC12558464; doi:10.1371/journal.pone.0334883)
Supplement: S1 Table — (DOCX) [file pone.0334883.s001.docx]

**Supplement**

S1: Comparison of characteristics of pediatric SCD Medicaid beneficiaries continuously enrolled and those with gaps the years 2017 – 2019

|  | CA | | | GA | | | MI | | | WI | | |
| --- | --- | --- | --- | --- | --- | --- | --- | --- | --- | --- | --- | --- |
|  | No gaps/  exits  (N = 1197) | With gaps  (N = 68) | p-value | No gaps/  Exits  (N = 1822) | With gaps  (N = 506) | p-value | No gaps/  exits  (N = 841) | With gaps  (N = 92) | p-value | No gaps/  exits  (N = 288) | With gaps  (N = 68) | p  -value |
| Gender |  |  | 0.1042 |  |  | 0.0026 |  |  | 0.1634 |  |  | 0.6536 |
| Female | 548  (45.8%) | 38  (55.9%) |  | 885  (48.6%) | 284  (56.1%) |  | 430  (51.1%) | 40  (43.5%) |  | 140  (48.6%) | 31  (45.6%) |  |
| Male | 649  (54.2%) | 30  (44.1%) |  | 937  (51.4%) | 222  (43.9%) |  | 411  (48.9%) | 52  (56.5%) |  | 148  (51.4%) | 37  (54.4%) |  |
| Disabled indivi-  -duals in 2017^a^ |  |  | 0.606 |  |  | <0.0001 |  |  | <0.0001 |  |  | - |
| Yes | 485  (41.0%) | 18  (26.4%) |  | 918  (50.4%) | 79  (15.6%) |  | 683  (81.2%) | 49  (53.3%) |  | 148  (51.4%) | <11 |  |
| No | 697  (59.0%) | 44  (64.7%) |  | 904  (49.6%) | 427  (83.9%) |  | 158  (18.8%) | 43  (46.7%) |  | 140  (48.6%) | >11 |  |

a: Disability plan information missing for 21 individuals from CA
