# Supplementary material for: Enrollment patterns among medicaid beneficiaries with sickle cell disease: Multistate findings from the sickle cell data collection program
Source: PLoS One. 2025 Oct 27;20(10):e0334883. doi: 10.1371/journal.pone.0334883 (PMC12558464; doi:10.1371/journal.pone.0334883)
Supplement: S2 Table — (DOCX) [file pone.0334883.s002.docx]

**Supplement**

S2: Comparison of characteristics of adult SCD Medicaid beneficiaries continuously enrolled and those with gaps the years 2017 – 2019

|  | CA | | | | GA | | | | MI | | | | WI | | |
| --- | --- | --- | --- | --- | --- | --- | --- | --- | --- | --- | --- | --- | --- | --- | --- |
|  | No gaps/  exits  (N = 2182) | With gaps  (N = 287) | p-value | No gaps/ exits  (N = 1606) | | With gaps  (N = 312) | p-value | No gaps/  exits  (N = 1491) | | With gaps  (N = 481) | p-value | No gaps/ exits  (N = 446) | | With gaps  (N=113) | p-value |
| Gender |  |  | 0.1137 |  | |  | <0.0001 |  | |  | 0.3353 |  | |  | 0.0081 |
| Female | 1419  (65.0%) | 173  (60.3%) |  | 1012  (63.0%) | | 254  (81.4%) |  | 963  (64.6%) | | 299  (62.2%) |  | 288  (64.6%) | | 58  (51.3%) |  |
| Male | 763  (35.0%) | 114  (39.7%) |  | 594  (37.0%) | | 58  (18.6%) |  | 528  (35.4%) | | 182  (37.8%) |  | 158  (35.4%) | | 55  (48.7%) |  |
| Disabled Indivi-  -duals in 2017^a^ |  |  | <0.0001 |  | |  | <0.0001 |  | |  | <0.0001 |  | |  | <0.0001 |
| Yes | 1373  (65.3%) | 75  (30.6%) |  | 1246  (77.6%) | | 74  (23.7%) |  | 1036  (69.5%) | | 193  (40.1%) |  | 343  (76.9%) | | 45  (39.8%) |  |
| No | 728  (34.7%) | 170  (69.4%) |  | 360  (22.4%) | | 238  (76.3%) |  | 455  (30.5%) | | 288  (59.9%) |  | 103  (23.1%) | | 68  (60.2%) |  |

a: disability information missing for 123 individuals from Ca
