# Supplementary material for: Enrollment patterns among medicaid beneficiaries with sickle cell disease: Multistate findings from the sickle cell data collection program
Source: PLoS One. 2025 Oct 27;20(10):e0334883. doi: 10.1371/journal.pone.0334883 (PMC12558464; doi:10.1371/journal.pone.0334883)
Supplement: S3 Table — (DOCX) [file pone.0334883.s003.docx]

**Supplement**

S3: Gaps in Medicaid enrollment among children with SCD

|  | CA (# gaps = 77) | GA (# gaps = 585) | | MI (# gaps = 101) | WI (# gaps = 88) |
| --- | --- | --- | --- | --- | --- |
| Rate of gaps per person | 1.13 | 1.16 | | 1.10 | 1.29 |
| Number (%) gaps less than a duration of 3 months | 19 (24.7%) | 360 (61.5%) | 43 (42.6%) | | 61 (69.3%) |
