# Supplementary material for: Enrollment patterns among medicaid beneficiaries with sickle cell disease: Multistate findings from the sickle cell data collection program
Source: PLoS One. 2025 Oct 27;20(10):e0334883. doi: 10.1371/journal.pone.0334883 (PMC12558464; doi:10.1371/journal.pone.0334883)
Supplement: S4 Table — (DOCX) [file pone.0334883.s004.docx]

**Supplement**

S4: Gaps in Medicaid enrollment among adults with SCD

|  | CA (# gaps = 347) | GA (# gaps = 370) | MI (# gaps = 604) | WI (# gaps = 168) |
| --- | --- | --- | --- | --- |
| Rate of gaps per person | 1.21 | 1.19 | 1.26 | 1.53 |
| Number (%) gaps less than a duration of 3 months | 143 (41.1%) | 193 (52.2%) | 249 (41.2%) | 87 (51.8%) |
